# Broadband Wind-Driven Hybrid Triboelectric–Electromagnetic Generator for Sufficient Self-Powered Atmospheric Environment Monitoring

**DOI:** 10.3390/mi17070809

**Published:** 2026-07-02

**Authors:** Shihan Zhang, Yidi Wang, Likun Gong

**Affiliations:** 1 American Heritage School, 12200 W Broward Blvd, Plantation, FL 33325, USA; corrinezsh@gmail.com; 2 State Key Laboratory of Heavy Oil Processing, China University of Petroleum-Beijing, Beijing 102249, China; 3 Beijing Key Laboratory of Oil and Gas Pollution Control, China University of Petroleum-Beijing, Beijing 102249, China; 4 College of Science, China University of Petroleum (East China), Qingdao 266580, China

**Keywords:** triboelectric–electromagnetic hybrids, broad-band wind energy, power management circuits, self-powered systems, atmospheric environment monitoring

## Abstract

Self-powered monitoring systems capable of scavenging ambient mechanical energy are a highly desirable solution to eliminate the reliance on batteries and grid power in remote and distributed atmospheric sensing networks. However, the widespread adoption of such systems is severely hindered by the insufficient output power density of current energy harvesters, which struggle to simultaneously drive environmental sensors, data acquisition units, and wireless transmission modules. In this work, we report a highly integrated hybrid power generation system that couples a triboelectric nanogenerator (TENG) and an electromagnetic generator (EMG) to efficiently harvest low-frequency mechanical energy from the surroundings. Through systematic structural optimization and synergistic matching of the two transduction mechanisms, the device achieves an outstanding volumetric power density of 129.9 W·m^−3^, which represents one of the highest values ever reported for hybrid nanogenerators targeting self-powered environmental applications. The output characteristics of both the TENG and EMG units under varying load impedances are thoroughly characterized, revealing the optimal operating points for maximum power extraction. A tailored power management module, consisting of rectification, energy storage, and regulation circuits, is designed to convert the irregular alternating output into a stable direct-current supply. To demonstrate the practical viability of the system, we construct a complete self-powered atmospheric environment monitoring node, which integrates multiple environmental sensors, a data acquisition module, and a wireless transmission module. Driven exclusively by the hybrid TENG–EMG generator under ambient mechanical excitation, the node successfully performs real-time sensing, signal processing, and remote data communication without any external power input. This work not only provides a record-high power density among hybrid generators for environmental monitoring, but also establishes a feasible pathway toward maintenance-free, widely distributed, and truly autonomous atmospheric sensing networks. The presented strategy of maximizing volumetric power density through hybrid design and impedance engineering can be readily extended to other self-powered systems.

## 1. Introduction

Atmospheric environmental quality is directly linked to human health and ecological safety. With rapid industrialization and urbanization, air pollution has become increasingly severe, creating an urgent demand for real-time monitoring of toxic and hazardous gases such as fine particulate matter (PM2.5), ammonia (NH_3_), nitrogen oxides (NOx), and volatile organic compounds (VOCs) [1,2,3]. Particularly in livestock farms, chemical industrial parks, landfill sites, and remote wilderness areas, the concentration and diffusion of pollutants exhibit high spatiotemporal variability and burstiness, necessitating the deployment of numerous sensing nodes to establish high-resolution environmental monitoring networks [4,5,6]. However, conventional environmental monitoring stations are bulky, costly, and dependent on grid power, making distributed deployment across vast areas challenging [7,8]. Although battery-powered portable sensors offer some flexibility, they face inherent limitations in outdoor and field scenarios, including finite battery life, difficult maintenance and replacement, and potential environmental contamination risks [9,10,11,12]. Consequently, developing a self-powered atmospheric monitoring system capable of autonomously harvesting energy from its surroundings—eliminating the need for external power sources and frequent maintenance—has emerged as a critical research direction in environmental sensing.

Among various ambient energy sources, wind energy stands out as an ideal candidate for powering outdoor sensing devices due to its wide distribution, renewability, cleanliness, and ubiquity [13,14,15]. However, natural wind is characterized by a broad speed range (1–12 m/s), low frequency, and strong stochasticity, posing significant challenges to the operating bandwidth, conversion efficiency, and environmental adaptability of energy harvesting devices [16,17,18]. Traditional electromagnetic generators (EMGs) exhibit high conversion efficiency in high-frequency regimes but deliver weak output under low-frequency, gentle breeze conditions, rendering them ineffective for harvesting dispersed ambient wind energy. In contrast, the recently emerging triboelectric nanogenerator (TENG) demonstrates distinct advantages in low-frequency mechanical energy harvesting. Its working mechanism, based on triboelectrification and electrostatic induction, endows it with high sensitivity to weak and irregular motions, making it particularly suitable for capturing low-speed wind energy [19,20,21]. Nevertheless, a single-mode TENG still falls short in power density and high-frequency response when confronted with highly fluctuating wind speeds or wide frequency spans.

To overcome the limitations of a single energy conversion mechanism, a hybrid generator strategy that synergistically integrates TENG and EMG has been proposed [22,23,24,25]. Through rational mechanical design, the TENG can effectively cover the low-frequency wind energy harvesting range, while the EMG delivers high power output under high wind speeds, achieving spectral complementarity and significantly broadening the overall system’s operational wind speed range [26,27]. Recent studies have explored TENG-EMG hybrid generators for self-powered environmental sensing. However, two critical challenges persist in practical wind energy harvesting: first, wide wind speed variations induce drastic fluctuations in generator output impedance and power, making it difficult for simple rectifier-storage circuits to maintain efficient energy utilization across the full wind speed spectrum; second, existing hierarchical power management strategies (such as isolated storage with undervoltage lockout) exhibit low efficiency in harnessing the high-power output of EMGs in high-frequency regimes, and complex circuit designs often introduce non-negligible energy losses [28,29]. These issues directly constrain the long-term, stable operation of self-powered atmospheric monitoring systems in real outdoor environments.

In light of these challenges, this work designs and fabricates a broadband wind energy-harvesting TENG-EMG hybrid generator (BW-TEHG) and develops a corresponding dual-mode power management technology (Dm-PMT), aiming to provide a fully self-powered energy solution for outdoor atmospheric environment monitoring—particularly for key indicators such as NH_3_ and PM2.5. The BW-TEHG integrates a vane-slapping structure freestanding-mode TENG and a sandwich-structured EMG via a dual-shaft coupling mechanism. The Dm-PMT intelligently switches between an “auto-wakeup” intermittent operation mode and a supercapacitor-based continuous power supply mode based on real-time wind speed and energy input status, thereby significantly enhancing the system’s ability to capture and utilize wind energy across a wide speed range (1–12 m/s). This study systematically optimizes the triboelectric materials, electrode grid number, and mechanical configuration of the TENG, as well as the coil arrangement of the EMG, and provides an in-depth analysis of their complementary output characteristics across a broad frequency band. Furthermore, we validate the feasibility of this self-powered system for driving atmospheric monitoring sensors and wireless transmission modules under simulated real wind conditions. The synergistic driving strategy of the BW-TEHG and Dm-PMT demonstrated herein paves a viable technical pathway for deploying maintenance-free, all-weather, in situ atmospheric environment monitoring nodes in diverse scenarios including farms, livestock facilities, industrial zones, and remote wilderness areas.

## 2. Experimental Section

### 2.1. Preparation of BW-TEHG

The TENG part utilized polytetrafluoroethylene (PTFE) elastic blades and copper (Cu) as triboelectric materials, specifically comprising four PTFE blade rotors and a stator with sixteen Cu grid electrodes. Each PTFE leaf measures 6 cm in length and 4 cm in width, evenly distributed on the rotor disk (with a 90° spacing). The gap between adjacent Cu electrodes is 1 mm, with an electrode width of 1.8 cm and a length of 6 cm. Sixteen Cu electrodes are arranged in an interleaved pattern on the stator disk, forming eight pairs of interdigitated electrode structures (i.e., an 8-grid structure). Among the eight pairs of Cu electrodes, the odd-numbered electrodes (1, 3, 5, 7, 9, 11, 13, 15) are connected in parallel as one output group, while the even-numbered electrodes (2, 4, 6, 8, 10, 12, 14, 16) are connected in parallel as another output group (Figure S1). For the EMG part, eight sets of parallel-connected copper coils (6 cm long, 3 cm wide) were fixed on a bottom annular support plate. Eight high-remanence permanent magnets were uniformly embedded in a circular rotor base and precisely positioned between the upper and lower coil layers via the drive shaft, forming a sandwich structure. A gap of 0.5 cm was maintained between the magnets and the coil surfaces.

### 2.2. Measurement and Characterization

A wind turbine was used to generate a wide range of wind speeds from 1 to 12 m/s. The rotational speeds of the TENG and EMG were measured using an infrared tachometer, with rotor speeds precisely controlled by a computer numerical control (CNC) motor. The microscopic morphology of the samples was characterized using a scanning electron microscope (Merlin Compact). The voltage, current, and other electrical output signals of the generators were measured and recorded using a Keithley 6514 electrometer.

## 3. Results and Discussion

### 3.1. Design of the Self-Powered Atmospheric Environment Monitoring System

**Figure 1:**
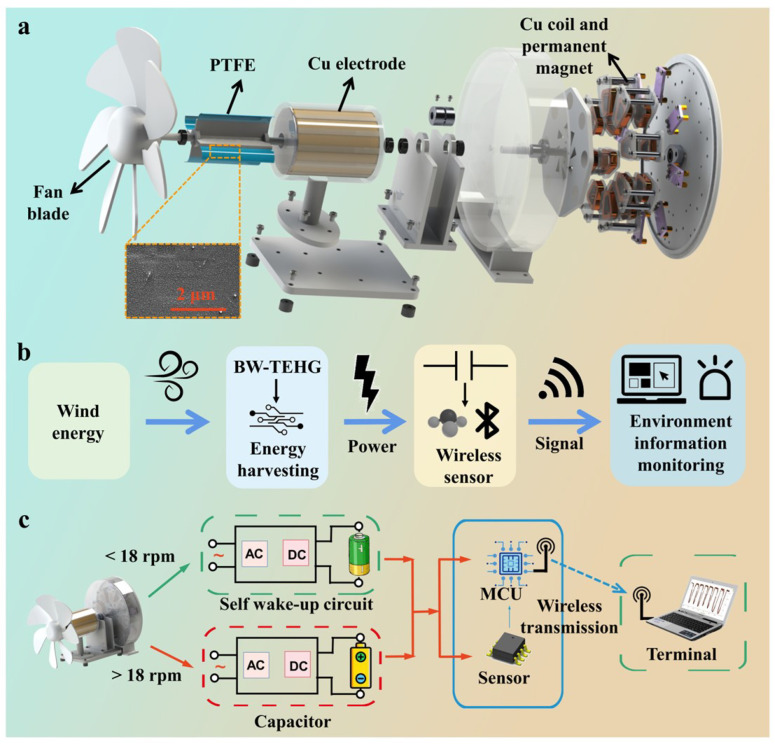
Design of the broadband wind-driven TENG-EMG hybrid generator (BW-TEHG). (**a**) Schematic illustration of the BW-TEHG structure. (**b**) Workflow diagram of the self-powered atmospheric environment monitoring system driven by wind energy. (**c**) Schematic diagram of the power management system for the BW-TEHG.

We designed and fabricated a compact (length × width × height: 35 cm × 18 cm × 22 cm) broadband wind-driven TENG-EMG hybrid generator (BW-TEHG). The system mechanically couples a vane-slapping structure TENG and a sandwich-structured EMG in a dual-shaft configuration. The working principle of the TENG is based on the coupling effect of triboelectrification and electrostatic induction, driven by sliding friction between a rotating PTFE film and interdigitated copper (Cu) electrodes. Scanning electron microscopy (SEM) images show that, under optimal fabrication conditions, the surface of the PTFE film is exceptionally smooth (Figure 1a), which helps to reduce frictional resistance and mechanical wear, thereby enhancing the long-term stability and operational lifespan of the device. When external wind drives the fan blades, the BW-TEHG converts mechanical energy into electricity, powering gas sensors, data acquisition circuits, and wireless transmission modules. The collected sensor signals are wirelessly transmitted to terminals such as computers, mobile phones, and the cloud, enabling real-time monitoring and early warning of atmospheric conditions (Figure 1b).

Given the low-frequency and chaotic nature of ambient wind energy, generating a stable power output directly for electronic devices is challenging. To address this, we specifically designed a dual-mode power management unit (Dm-PMT). This unit first converts AC to DC via rectification and voltage regulation circuits, enabling efficient wind energy harvesting and storage across a wide wind speed range (1–12 m/s). When the wind speed exceeds 3 m/s (corresponding to a system rotational speed above 18 rpm), a supercapacitor stores the electrical energy generated by the BW-TEHG. Conversely, when the wind speed falls below 3 m/s (speed < 18 rpm) or the supercapacitor’s stored energy is insufficient, the system activates an “auto-wakeup” mode for intermittent power supply, ensuring basic continuity of gas monitoring and data transmission. The functional complementarity of the TENG and EMG output characteristics, coupled with the synergistic action of the Dm-PMT, effectively compensates for output instability caused by wind speed fluctuations (Figure 1c).

### 3.2. Optimization and Performance Testing of the TENG

**Figure 2:**
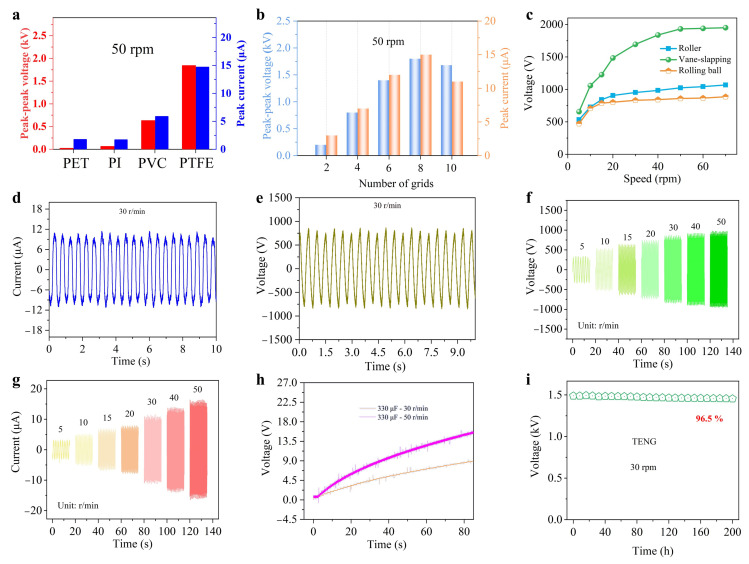
Performance testing of the TENG. Electrical output of the TENG with (**a**) different triboelectric materials, (**b**) different numbers of grating electrodes, and (**c**) different structures. (**d**) Short-circuit current (Isc) and (**e**) open-circuit voltage (Voc) characteristics of the TENG at a rotational speed of 30 rpm. (**f**) Voc and (**g**) Isc of the TENG at different rotational speeds. (**h**) Voltage curves of a capacitor charged by the TENG at different rotational speeds. (**i**) Durability of the Voc of the TENG.

The TENG employed in this study operates in freestanding mode. While keeping the stator (Cu electrodes) unchanged, we investigated the influence of different triboelectric materials (PET, PI, PVC, PTFE) covering the rotor surface on electrical output performance. Results indicate that the PTFE film, owing to its superior electron affinity, exhibits the highest electrical output and was therefore selected for subsequent experiments (Figure 2a). At 50 rpm, the peak-to-peak transferred charge of the PTFE-based TENG reached 1.8 μC, the highest among the four materials (Figure S2). Data were smoothed using the Savitzky–Golay filter (OriginPro 2021, window points: 15, polynomial order: 2) to remove high-frequency noise for clearer visualization. The smoothing does not alter the peak charge values or the comparative trends. The surface charge density of different triboelectric materials was further calculated. The total effective contact area between the four PTFE pieces and Cu was approximately 0.012 m^2^. The results show that PTFE achieves the highest surface charge density of 150.1 μC/m^2^, consistent with its output performance trend, further confirming the excellent triboelectric properties of PTFE (Figure S4). We tested the electrical output of TENGs made of PTFE films with thicknesses of 50, 100, 200, and 300 μm. The results show that output performance first increases and then stabilizes with increasing thickness. This is mainly because a moderate thickness improves charge retention, while excessive thickness reduces flexible contact and effective contact area, thus limiting output enhancement. PTFE films of 200 μm thickness were used in subsequent experiments.

Under a fixed rotational speed of 50 rpm, the effect of the number of grating electrodes on TENG output was studied. As the number of gratings gradually increased from 2 pairs to 8 pairs, both the open-circuit voltage (Voc) and short-circuit current (Isc) of the TENG increased significantly. This is attributed to the reduced size of individual electrode units with increasing grating number, improving the matching between the electrode structure and the friction interface, leading to a more uniform interfacial electric field distribution and promoting directional charge migration and efficient separation. Simultaneously, the increased number of charge induction cycles per unit time enhances the overall transfer rate. However, when the number of gratings increased to 10 pairs, the electrical output declined. This is because excessive gratings increase the proportion of electrode gaps, intensifying boundary electric field distortion and weakening the charge driving force. Concurrently, the effective area of a single grating is excessively compressed, and the reduction in accumulated charge per unit cannot be compensated by the increase in quantity. Considering factors such as charge density, electric field uniformity, and effective unit area comprehensively, the 8-grid structure was determined as the optimal choice for the current system (Figure 2b).

Regarding structural topology optimization, we compared the peak voltage output characteristics of three typical structures—vane-slapping, rolling-ball, and roller structures—under the same material system. The detailed structural schematic diagram of the three types of TENG structures is shown in Figure S5. The vane-slapping structure rotor consists of 4 sliding friction blades. The rolling-ball structure rotor is composed of 6 PTFE-wrapped rolling balls, while the roller structure rotor is made up of 6 PTFE-wrapped rolling cylinders. All stators are composed of interdigitated electrodes, with each electrode featuring 8 grids. Results show that the vane-slapping structure consistently exhibits the highest output voltage across all tested speeds. This superiority primarily stems from its dominant sliding friction mode, which, compared to rolling modes, involves stronger interfacial shear forces and a more intense contact–separation process, favoring the induction of higher-density surface charge accumulation (Figure 2c).

The TENG corresponds to the typical wind speed (3.5 m/s) in natural wind environment at a rotational speed of 30 rpm. At a rotational speed of 30 rpm, the Isc and Voc waveforms of the TENG demonstrate good periodicity and uniformity (Figure 2d,e). The minimum starting speed of the TENG is 5 rpm. As the speed increases from 5 rpm to 50 rpm, the peak Voc rises from 343 V to 966 V, and the peak Isc increases from 1.06 μA to 5.29 μA (Figure 2f,g). When the speed exceeds 50 rpm, the output stabilizes, indicating that charge accumulation induced by electrostatic induction reaches saturation. At this point, the rate of charge generation reaches a dynamic equilibrium with air breakdown, charge leakage, and interface recombination rates. Therefore, further increasing the rotational speed will not significantly increase the amount of surface accumulated charge, leading to a stable output voltage. Charging tests on a 330 μF capacitor show that at 30 rpm and 50 rpm, the capacitor voltage reaches 9 V and 15 V, respectively, within 80 s (Figure 2h). Furthermore, durability testing after 200 h of continuous operation shows that the peak-to-peak Voc remains at 96.5% of its initial value, confirming the excellent long-term operational reliability of the TENG (Figure 2i).

### 3.3. Optimization and Performance Testing of the EMG

**Figure 3:**
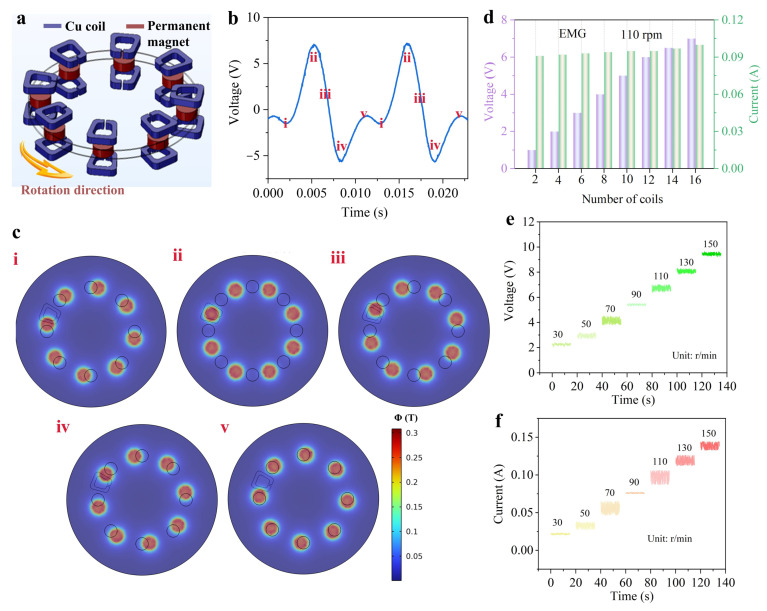
Performance testing of the EMG. (**a**) Schematic diagram of the 3D EMG model established in COMSOL. (**b**) Electromotive force of the EMG over a complete cycle (i–v) calculated by COMSOL finite element simulation, and (**c**) magnetic flux within the coil at corresponding displacement positions. (**d**) Electrical output of the EMG with different numbers of coils. (**e**) Voc and (**f**) Isc of the EMG at different rotational speeds.

To intuitively reveal the electromagnetic field changes during EMG operation, we performed finite element simulations using COMSOL Multiphysics software (version 5.3). Driven by wind, high-remanence permanent magnets fixed at the edge of the rotor disk rotate periodically between the upper and lower layers of copper coils, continuously cutting magnetic field lines and causing the magnetic flux through the coils to alternate. According to Faraday’s law of electromagnetic induction, the changing magnetic flux induces an electromotive force across the coil terminals (Figure 3a). Simulation results show that when the magnet completely covers the coil area or is far away from it, the rate of magnetic flux change is minimal, and the induced EMF is near zero (state i). As the edge of the magnet sweeps across the effective coil area, the magnetic flux changes rapidly, and the induced EMF reaches a peak (states ii, iii). As the magnet moves further away, the rate of flux change decreases, and the output voltage subsequently decays (state iv), thus forming a periodic AC signal (Figure 3b,c). Although the magnetic flux density maps appear similar at the five locations, the rates of magnetic flux change differ at different locations. The peak values of induced electromotive force occur in the regions where the magnetic flux changes most dramatically (states ii and iv), while the induced electromotive force is zero at the extreme points of magnetic flux (states i and v).

For the EMG composed of two layers of coils, we investigated the effect of the number of series-connected coils on its electrical output. Eight coil configurations, from 2 × 1 to 2 × 8, were tested. Results indicate that as the number of series-connected coils increases, the rectified voltage gradually rises, while the current remains nearly constant. According to Faraday’s law, more coils in series accumulate the induced electromotive force, thereby boosting the total output voltage. However, the increased internal resistance of the coils limits further current enhancement. Considering size constraints and output performance, we selected the 2 × 8 configuration (total of 16 coils) for subsequent experiments (Figure 3d). As shown in Figure 3e,f, both Voc and Isc of the EMG exhibit a good linear positive correlation with rotational speed. As the speed increases from 30 rpm to 150 rpm, Voc rises from 2.35 V to 9.64 V, and Isc increases from 0.023 A to 0.144 A, consistent with the description of Faraday’s law.

### 3.4. Optimization and Performance Testing of the TENG-EMG Hybrid Generator

**Figure 4:**
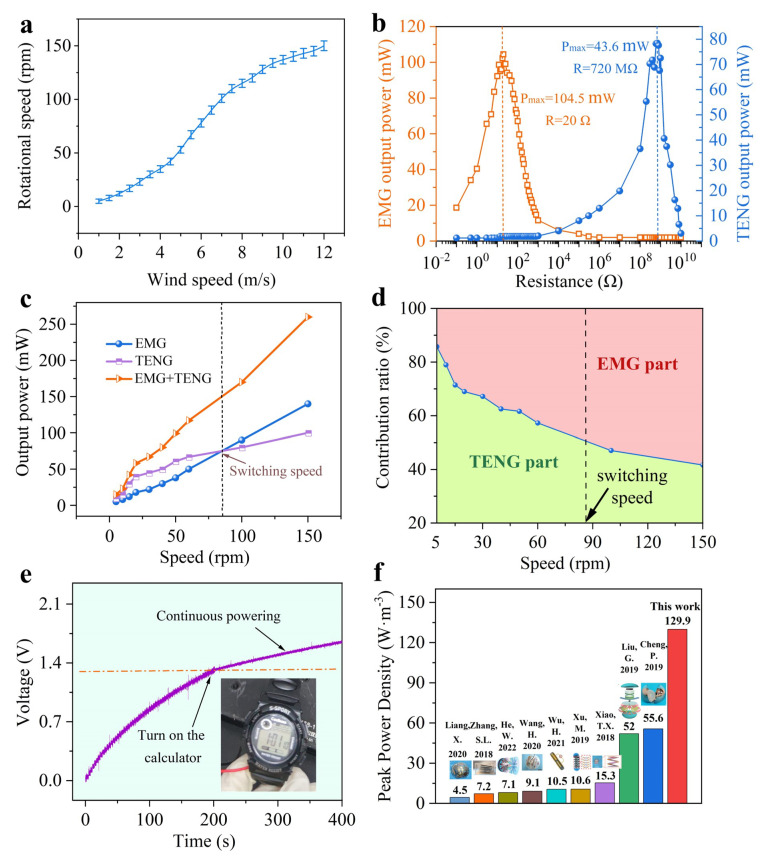
Performance evaluation of the BW-TEHG. (**a**) Rotational speed of the BW-TEHG under different wind speeds. (**b**) Relationship between peak output power of the TENG and EMG and different load resistances. (**c**) Output power of the generators at different rotational speeds. (**d**) Contribution ratio of TENG and EMG power generation under different wind speeds. (**e**) Voltage curve of the BW-TEHG charging a capacitor to power a digital watch. (**f**) Comparison of peak power density between this work and other previously published works [30,31,32,33,34,35,36,37,38].

We first measured the rotational speed variation curve of the BW-TEHG system under different wind speeds, and the results indicate a good linear relationship within the tested range (Figure 4a). Figure 4b illustrates the output power characteristics of the TENG and EMG under different load resistances. The TENG achieves a maximum output power of 43.6 mW at an external resistance of 720 MΩ, while the EMG achieves 104.5 mW at 20 Ω (detailed impedance matching tests are shown in Figures S6 and S7). Among them, TENG and EMG were measured at 30 rpm and 120 rpm, respectively.

Comparative analysis of the output power of the two generators at different rotational speeds reveals significant complementary characteristics (Figure 4c). When the rotational speed is below 80 rpm, the output performance of the TENG is markedly superior to that of the EMG, as the triboelectrification process remains effective at low speeds, whereas the electromagnetic induction effect is weak due to the low rate of magnetic flux change. As the rotational speed further increases, the output power of the EMG grows rapidly and gradually becomes dominant. This complementary mechanism is the core of the hybrid generator’s broadband response capability. Figure 4d quantitatively illustrates the contribution ratios of the TENG and EMG to the total system power at different wind speeds, clearly revealing the TENG’s dominant role in the low wind speed regime and the EMG’s advantage at high wind speeds.

In practical application testing, at a rotational speed of 30 rpm, the BW-TEHG charged a 1 mF capacitor to 1.33 V in just 194 s, successfully powering a digital watch continuously (Figure 4e). At 150 rpm, the BW-TEHG fabricated in this work achieved a high volumetric power density of 129.9 W·m^−3^, demonstrating a significant advantage compared to previously reported similar works (Figure 4f) [30,31,32,33,34,35,36,37,38]. Detailed information regarding the output power and volume of TENG and EMG can be found in Table S2.

### 3.5. Dual-Mode Power Management and Validation of Self-Powered Sensing Applications

**Figure 5:**
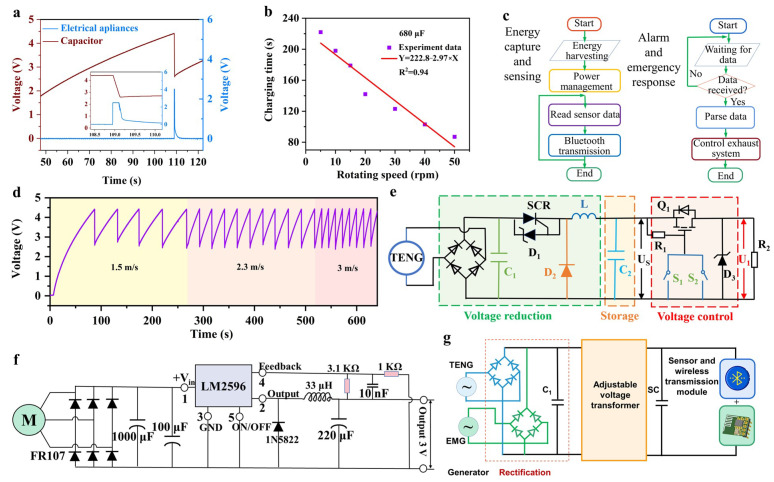
Power management circuit and testing of the BW-TEHG. (**a**) Comparison between the discharge moment of the storage capacitor and the microcontroller terminal voltage. (**b**) Discharge time of the auto-wakeup circuit at different rotational speeds. (**c**) Flowchart of the self-powered atmospheric environment monitoring. (**d**) Voltage of the storage capacitor in the auto-wakeup system under different wind speeds. (**e**) Schematic diagram of the auto-wakeup circuit principle when powered by the TENG. (**f**) Power management circuit for the EMG. (**g**) Schematic diagram of the power management circuit when powered by the BW-TEHG.

The incorporation of Dm-PMT significantly extends the practical wind speed range of the BW-TEHG. The system operates in two modes: at low wind speeds (1–3 m/s), power is primarily generated by the TENG, and energy is accumulated and released via the “auto-wakeup” module. When the voltage of the storage capacitor reaches the startup threshold of 4.4 V, the detection system is activated and operates for 200 milliseconds to complete data acquisition and transmission, then enters the next charging cycle (Figure 5a). Tests show that as the rotational speed increases from 10 rpm to 50 rpm, the time required for the storage capacitor to reach the threshold voltage decreases significantly, down to approximately 80 s, validating the system’s effective response capability across a wide speed range (Figure 5b). Figure 5c illustrates the system workflow and alarm triggering mechanism. At wind speeds of 1.5 m/s, 2.3 m/s, and 3 m/s, the 680 μF storage capacitor completes a full charge–discharge cycle in 45 s, 24 s, and 13 s, respectively (Figure 5d). Figure 5e presents a schematic diagram of the TENG-based auto-wakeup circuit. The circuit consists of a rectifier circuit, a voltage step-down circuit, and a voltage control circuit. The rectifier circuit converts alternating current (AC) into direct current (DC). The voltage step-down circuit reduces the voltage and increases the current, thereby improving the charging efficiency. The core function of the voltage control circuit is as follows: when the output voltage of the TENG is relatively low, the energy is first stored in the storage capacitor C_1_. Once the voltage across C_1_ reaches the preset threshold voltage (4.4 V), the circuit is triggered to conduct, supplying power to the MCU and sensors. After completing one cycle of data acquisition and transmission, the circuit automatically disconnects and enters the next charging cycle. The detailed circuit principles and operation are as follows. In the circuit, TPS3839 voltage monitoring chips are used to control the turning on and off of S1 and S2. For more detailed circuit principles and device selection, please refer to the work by Qin et al. and its Supplementary Materials [39].

**Figure 6:**
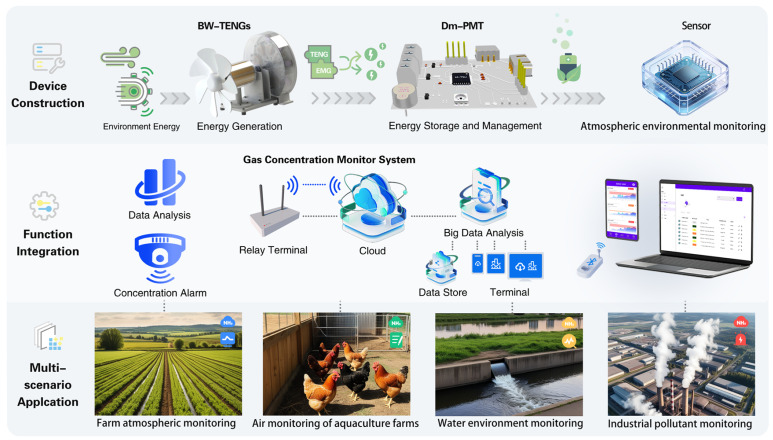
Application prospect diagram of the wind energy self-powered atmospheric environment monitoring system.

When the wind speed exceeds 3 m/s (rotational speed > 18 rpm), the electrical energy generated by both the TENG and EMG is managed by the power management module (Figure 5f), rectified, and regulated to provide a continuous and stable DC power supply for the downstream atmospheric monitoring sensors and wireless data transmission module (Figure 5g). At a wind speed of 4–5 m/s, the BW-TEHG system generates sufficient energy to maintain continuous operation of the data transmission module (Supporting Video S1). The video is played at double speed. Simultaneously, electrical energy is stored in a supercapacitor for backup use. Using a smartphone as a controller, data signals are received via Bluetooth, and the NH_3_ concentration variation curve is displayed on the screen. Figure S8 provides schematic diagrams of the power management module. In the video, we use a commercial wireless resistor acquisition module (LinkZill 01RC) as a tool for ammonia sensor data acquisition and wireless transmission demonstration. The power supply of the LinkZill 01RC module is provided by BW-TEHG through a rectifier energy storage circuit, which includes capacitors for storing electrical energy. The system does not have a built-in battery. Table S1 lists the operating voltage, current, power consumption, and duration of key components such as the MCU, Bluetooth module, and NH_3_ sensor, clearly illustrating the energy allocation of the system under self-powered conditions. The peak power of the system is approximately 87 mW, and in the self-wake-up intermittent mode, it operates for only 500 ms per cycle, resulting in a significantly reduced average power consumption. Looking forward, this system demonstrates excellent performance in utilizing broadband wind energy, ensuring stable atmospheric environment detection. Before practical application, this system must also undergo long-term outdoor deployment testing, which involves complex environmental factors and long-term reliability evaluation. The overall system framework is illustrated in Figure 6. The highly adaptable self-powered atmospheric environment monitoring system enables in situ self-powered atmospheric monitoring in various scenarios—such as farm air quality monitoring, chicken farm air monitoring, water body pollutant monitoring, and industrial pollution surveillance—presenting a promising pathway for addressing current global energy and environmental crises.

## 4. Conclusions

This study successfully designed, optimized, and validated a broadband wind energy harvesting TENG-EMG hybrid generator (BW-TEHG) and its dual-mode power management system (Dm-PMT) for constructing a fully self-powered atmospheric environment monitoring platform. Through systematic experimental optimization, the optimal configuration for the TENG unit was determined to be a vane-slapping structure with PTFE as the triboelectric material and 8-grid Cu electrodes, achieving a peak power of 43.6 mW in the low-speed region (<80 rpm). Simultaneously, the optimized 2 × 8 coil sandwich-structured EMG delivered a peak power of 104.5 mW in the high-speed region. Their dual-shaft coupling enabled complementary energy harvesting across a wide wind speed range. The designed Dm-PMT intelligently switches power supply paths based on wind speed: in the low-wind-speed regime (1–3 m/s), it relies on the TENG-driven “auto-wakeup” circuit for intermittent monitoring and data transmission; in the high-wind-speed regime (>3 m/s), the TENG and EMG synergistically provide continuous and stable power, effectively addressing system endurance under low energy input while ensuring real-time monitoring continuity. The constructed BW-TEHG achieved a peak volumetric power density of 129.9 W·m^−3^ and successfully powered NH_3_ gas sensing and wireless data transmission modules, providing an efficient and reliable energy solution for deploying maintenance-free, all-weather, self-powered atmospheric environment monitoring nodes in distributed scenarios such as smart agriculture, industrial emission monitoring, and remote wilderness ecological in situ monitoring.

## Data Availability

The data presented in this study are available on request from the corresponding author.

## Supplementary Materials

The following supporting information can be downloaded at https://www.mdpi.com/article/10.3390/mi17070809/s1, Figure S1: Schematic diagram of TENG’s structural parameters; Figure S2: Transfer charge of TENG in four media: PET, PI, PVC, and PTFE; Figure S3: Surface charge density of four materials: PET, PI, PVC, and PTFE; Figure S4: Open-circuit voltage of PTFE with different thicknesses; Figure S5: (a) shows the structural schematic of the vane-slapping TENG, (b) the rolling-ball mode TENG, and (c) the roller mode TENG; Figure S6: Output voltage and current of TENG under different matching impedances; Figure S7: EMG output voltage and current under different matching impedances; Figure S8: Photo of power management module; Table S1: Detailed power budget table; Table S2: Summary table of energy and power density; Video S1: Self-powered ammonia concentration monitoring system.
