# Supplementary material for: Broadband Wind-Driven Hybrid Triboelectric–Electromagnetic Generator for Sufficient Self-Powered Atmospheric Environment Monitoring
Source: Micromachines (Basel). 2026 Jul 2;17(7):809. doi: 10.3390/mi17070809 (PMC13414362; doi:10.3390/mi17070809)
Supplement: Supplementary file 1 [file micromachines-17-00809-s001.zip › Supporting information.pdf]

Broadband Wind-Driven Hybrid Triboelectric–Electromagnetic Generator for Sufficient Self-Powered Atmospheric Environment Monitoring

Shihan Zhang [1], Yidi Wang [2,3,*] and Likun Gong [4,*]

[1] American Heritage School, 12200 W Broward Blvd, Plantation, FL 33325, USA; corrinezsh@gmail.com
[2] State Key Laboratory of Heavy Oil Processing, China University of Petroleum-Beijing, Beijing 102249, China
[3] Beijing Key Laboratory of Oil and Gas Pollution Control, China University of Petroleum-Beijing, Beijing 102249, China
[4] College of Science, China University of Petroleum (East China), Qingdao 266580, China

* Correspondence: 2023880028@cup.edu.cn (Y.W.); gonglikun@foxmail.com (L.G.)

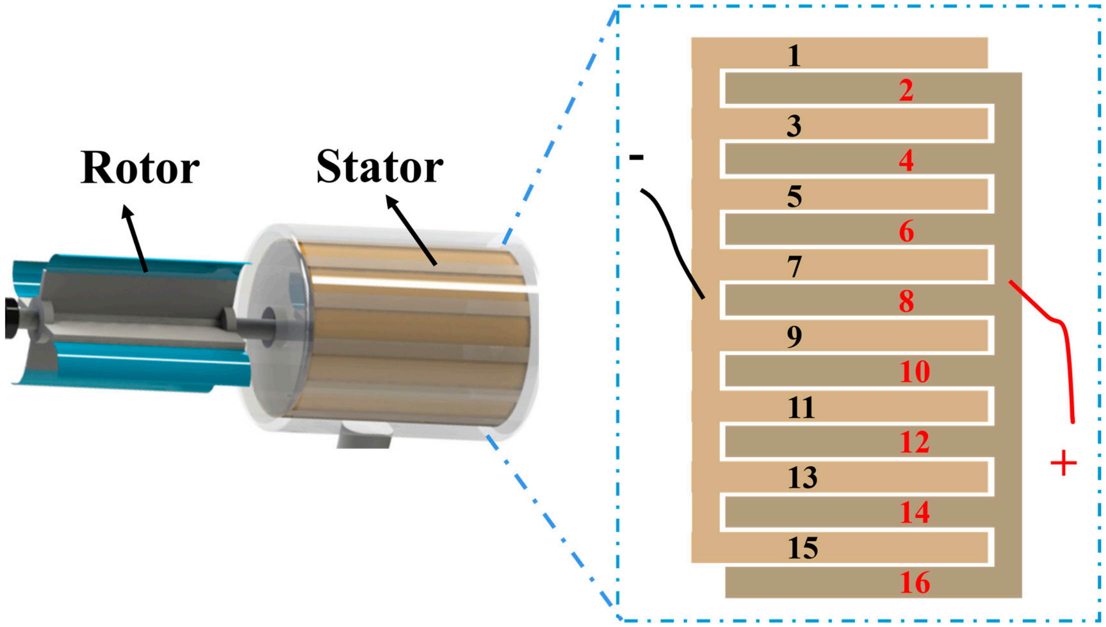

Figure S1. Schematic diagram of TENG's structural parameters.

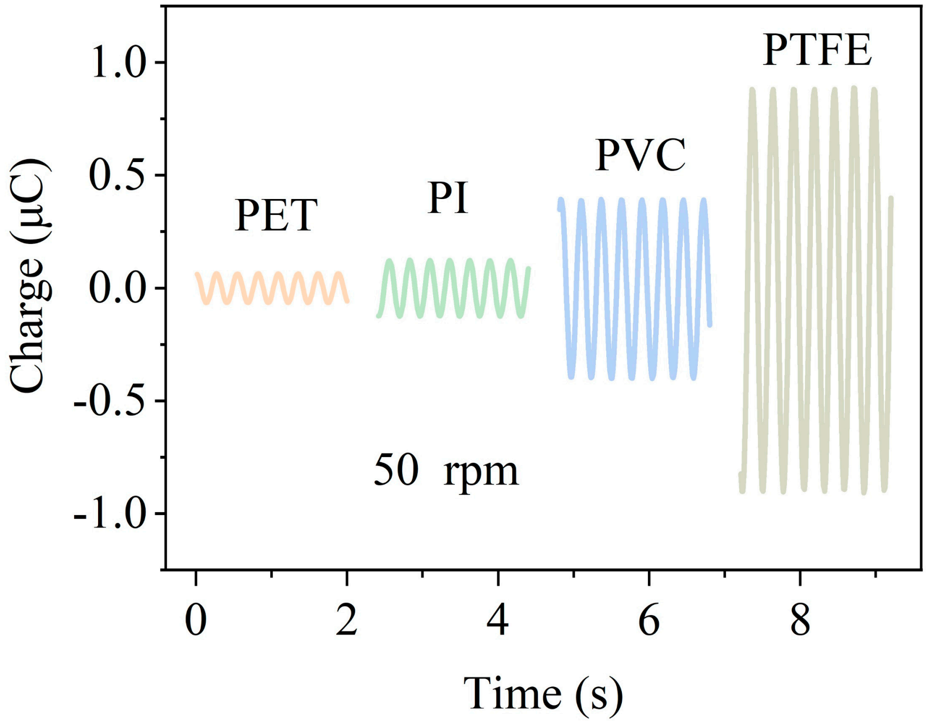

Figure S2. Transfer charge of TENG in four media: PET, PI, PVC, and PTFE.

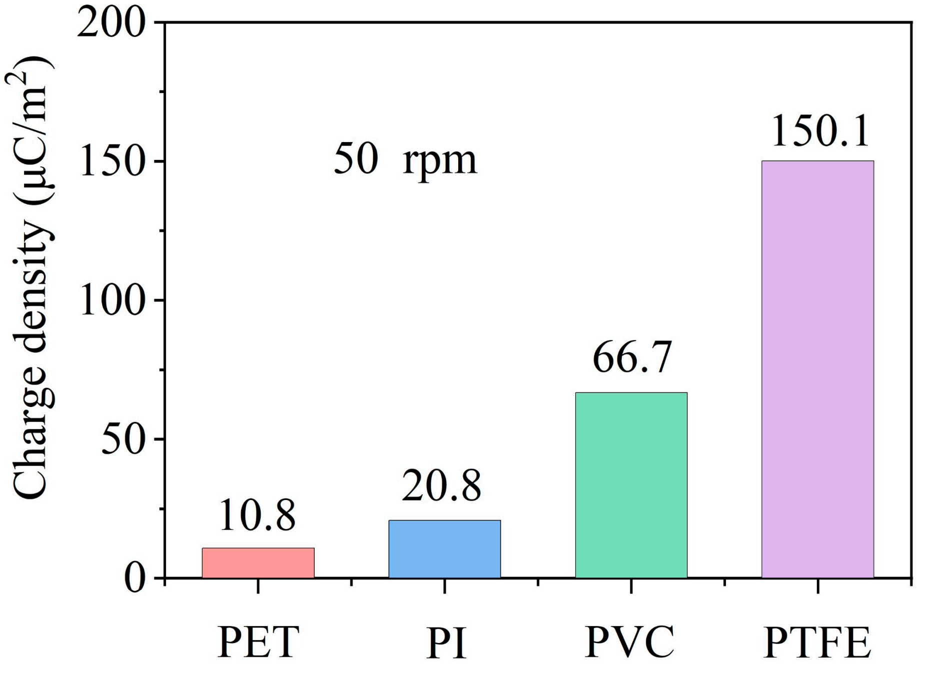

Figure S3. Surface charge density of four materials: PET, PI, PVC, and PTFE.

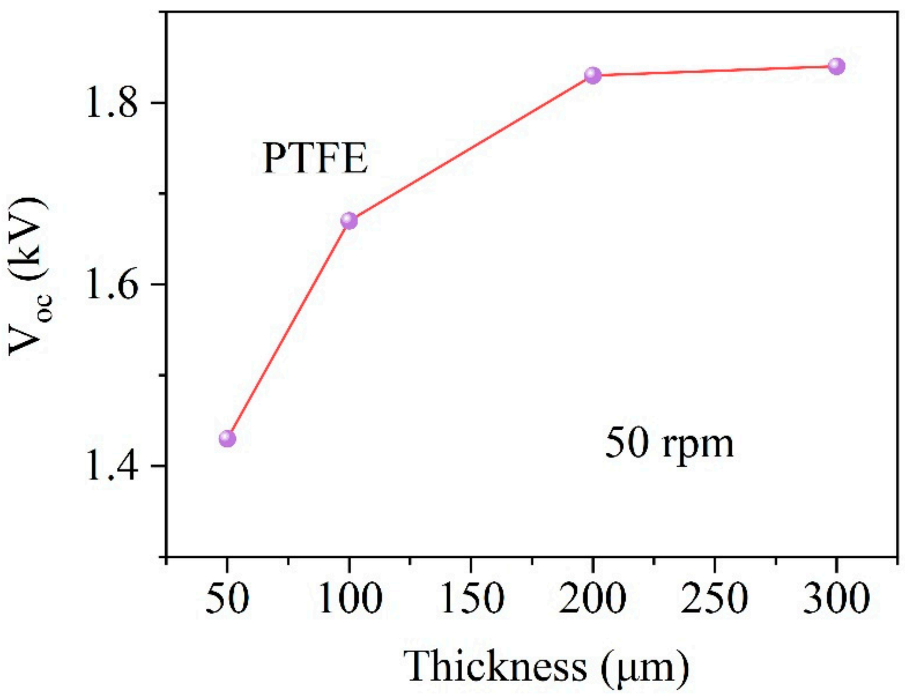

Figure S4. Open-circuit voltage of PTFE with different thicknesses.

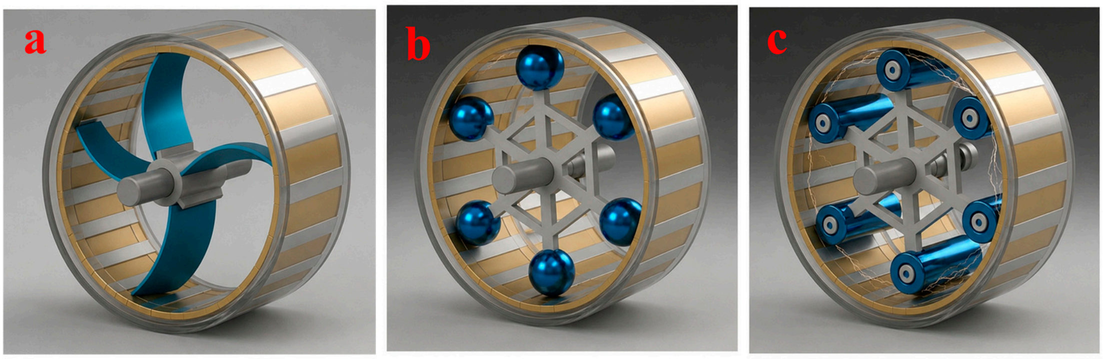

Figure S5. (a) shows the structural schematic of the vane-slapping TENG, (b) the rolling-ball mode TENG, and (c) the roller mode TENG.

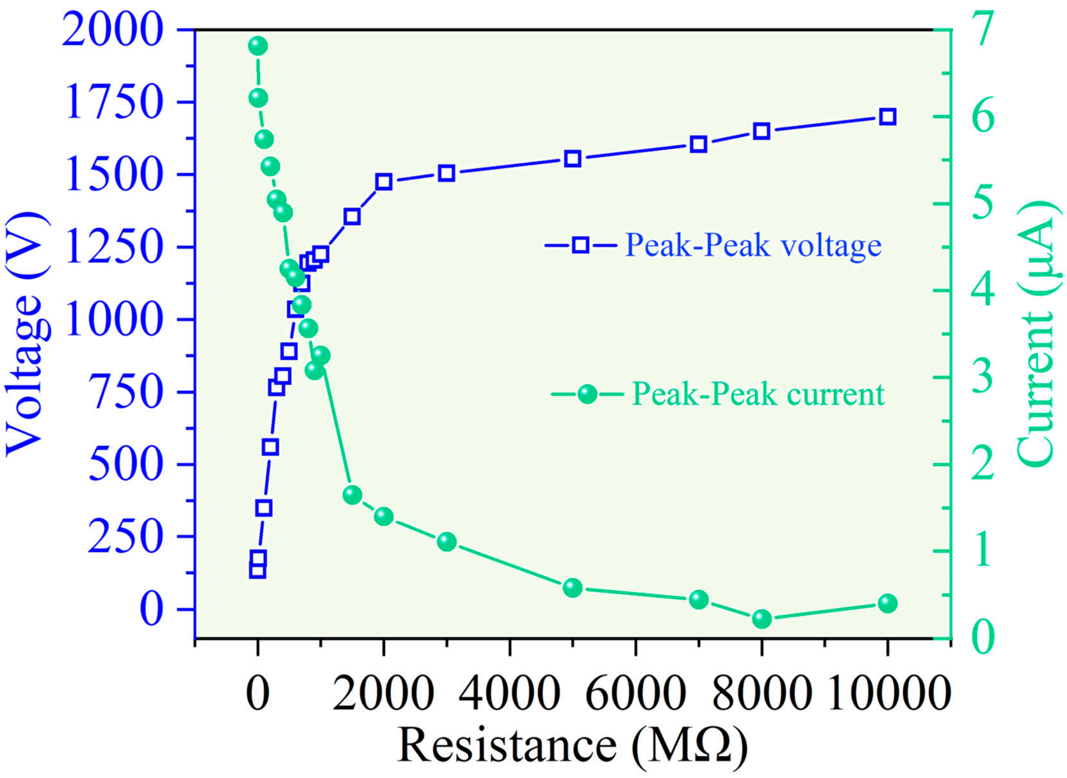

Figure S6. Output voltage and current of TENG under different matching impedances.

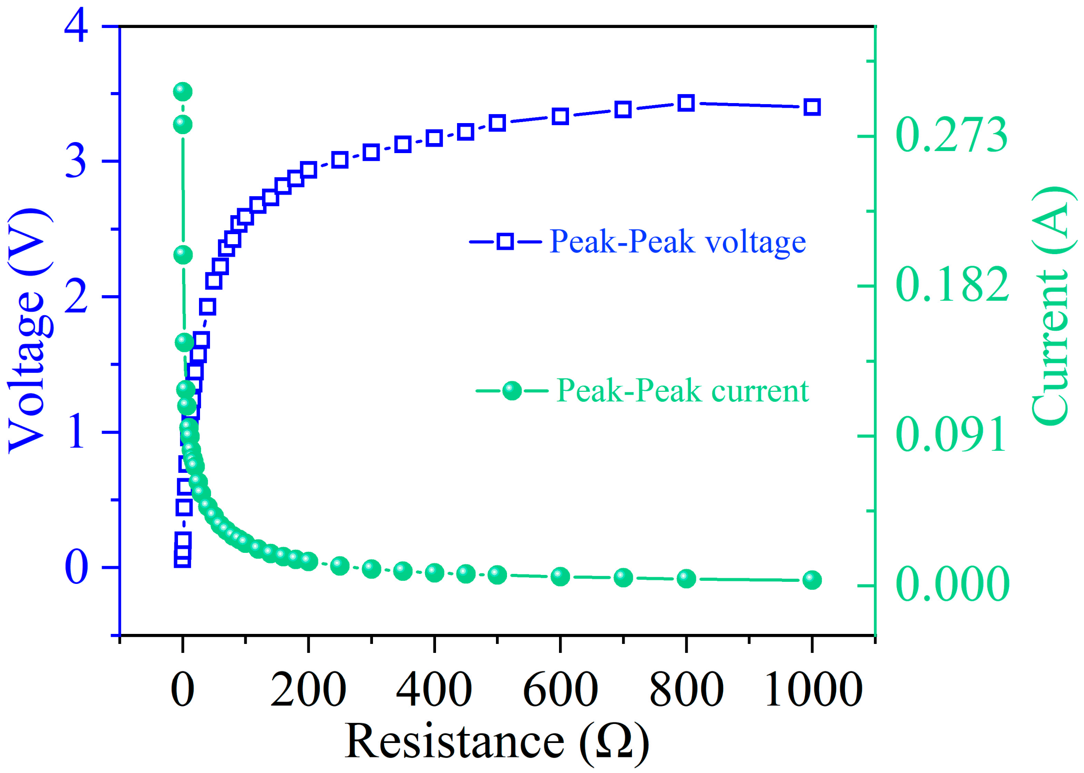

Figure S7. EMG output voltage and current under different matching impedances.

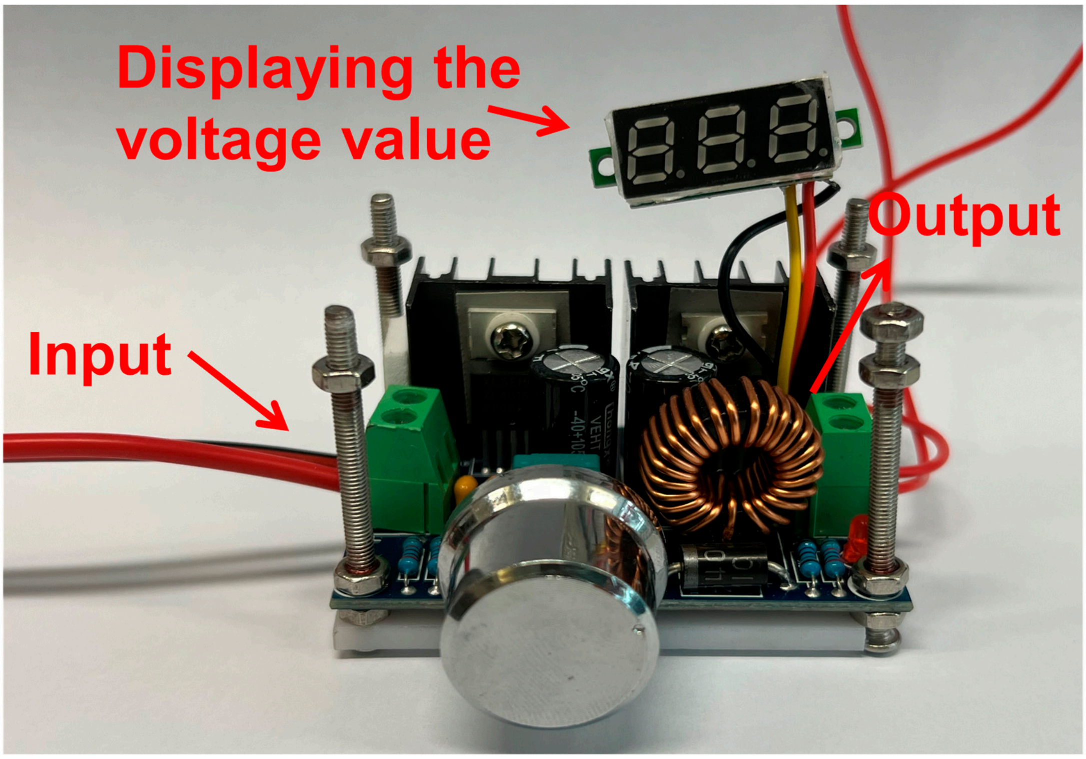

Figure S8. Photo of power management module.

Table S1. Detailed power budget table.

Component	Operating voltage (V)	Dormant current (μA)	Operating current (mA)	Single working time (ms)	Single work energy consumption (μJ)	Peak power consumption (mW)
MCU	3.3	0.8	4.2	500	6.93	13.86
Bluetooth module	3.3	1.2	4.8	500	7.92	15.84
NH_3 sensor	3.3	0.1	12	300	19.8	39.6
Signal conditioning and ADC	3.3	0.5	1.0	200	0.66	3.3
Voltage regulator and power management loss (estimated at 20%)	-	-	-	-	-	14.52
Total	-	-	-	-	35.31 μJ/time	87.12 mW

Table S2. Summary table of energy and power density.

Parameter	TENG	EMG	hybrid system
Volume (dm^3)	0.785	1.065	1.85
Peak power（mW）	100	140	240
Volumetric power density (W/m^3)	127.4	131.5	129.9
Single-lap energy (mJ)	40	56	96
Volumetric energy density (J/m^3)	50.9	52.6	51.9
